# Ultrasound-guided needle positioning confirmation with injection of saline solution for nodal dynamic contrast-enhanced MR-lymphangiography in pediatric patients

**DOI:** 10.1007/s00330-025-11346-1

**Published:** 2025-01-15

**Authors:** Julia Wagenpfeil, Katharina Hoß, Andreas Henkel, Sergej Geiger, Julian Alexander Luetkens, Daniel Kuetting, Claus Christian Pieper

**Affiliations:** Division for Minimally-invasive Lymph Vessel Therapy, Department of Diagnostic and Interventional Radiology, University Hospital of Bonn, Bonn, Germany

**Keywords:** Magnetic resonance lymphangiography, Ultrasound, Lymphatic imaging, Pediatric imaging

## Abstract

**Purpose:**

To assess the success rate of confirmation of ultrasound-guided intranodal needle positioning by saline injection for dynamic contrast-enhanced magnetic resonance lymphangiography (DCMRL) in pediatric patients.

**Material and methods:**

Data from children undergoing nodal DCMRL after ultrasound-guided needle positioning into inguinal lymph nodes and validation of the needle position by injection of plain saline solution between 05/2020 and 12/2022 were reviewed. On injection of saline solution, adequate needle position was confirmed by lymph node distension without leakage. Detection rates and lymph node diameters were recorded. Technical success of needle placement was defined as enhancement of ipsilateral iliac draining lymph vessels on DCMRL, while clinical success was based on enhancement of central lymphatics and/or lymphatic pathologies being observed.

**Results:**

One hundred sixteen DCMRL were performed in 90 children (58 male, mean age 6.1 years, range 3 weeks–18 years). In 232/232 groins lymph nodes were identified on ultrasound with a mean diameter of 2.5 mm (smallest diameter 1 mm in *n* = 24 patients). Due to skin inflammation, no puncture was performed in 2/232 groins. Saline injection showed lymph node distension in 230/230 punctured nodes. On MR contrast injection enhancement of draining lymph vessels was seen in 228/230 nodes. In 1/230 nodes, in-bore needle retraction led to lymphatic enhancement, so a total of 229/230 needle placements were technically successful. One of the 230 DCMRLs was successful with only a unilateral contrast application. Overall, 116/116 DCMRLs were clinically successful.

**Conclusion:**

Confirmation of ultrasound-guided needle positioning for nodal DCMRL using saline injection is a reliable technique with a very high success rate in pediatric patients with small lymph nodes.

**Key Points:**

***Question*** *Evaluation of ultrasound-guided injection needle positioning for dynamic contrast-enhanced MR-lymphangiography in children requires validation*.

***Findings*** *Confirmation of needle positioning by ultrasound-guided saline injection is a reliable technique with a very high success rate for MR-lymphangiography in pediatric patients*.

***Clinical relevance*** *Intranodal needle position for dynamic contrast-enhanced lymphangiography can be confirmed with a very high success rate using saline injection alone in a pediatric cohort*.

## Introduction

The spectrum of lymphatic imaging and interventional treatment options for lymphatic diseases has grown rapidly in recent years and has expanded to applications in very young patients [1]. For many years, lymphatic imaging was confined to transpedal fluoroscopic lymphangiography and lymphoscintigraphy. Subsequently, the introduction of nodal fluoroscopic lymphangiography with ultrasound-guided lymph node access has increased the range of applications of lymphatic imaging and interventions [1, 2]. Nevertheless, fluoroscopic lymphangiography is generally linked to potential complications arising from the use of an oil-based contrast agent, as well as ionizing radiation exposure [2, 3]. For purely diagnostic purposes, magnetic resonance lymphangiography (MRL) therefore provides a less invasive alternative and can be carried out with either nodal or interstitial transpedal contrast-agent application [4–6]. Nodal dynamic contrast-enhanced magnetic resonance lymphangiography (DCMRL) in particular plays an increasingly important role in the diagnostic work-up of patients with suspected central lymphatic abnormalities (e.g., chylothorax or plastic bronchitis) but is logistically and technically challenging [7]. The most important technical factor for a successful DCMRL examination is a stable nodal needle position used for contrast application during the examination [8]. As ultrasound-guided needle placement must be performed outside of the magnetic field of the MR scanner, confirmation of such a needle position prior to the actual MR examination is of utmost importance to avoid unnecessary patient transfers and unacceptably long examination times.

To this end, various techniques including fluoroscopic needle position confirmation by injection of iodine-based contrast agent, as well as sonographic confirmation either by injection of ultrasound contrast agent or plain saline solution have been proposed [1, 8, 9]. The latter technique is the simplest technically and does not rely on specialized equipment, e.g., an Angio-MR-Suite (XMR) or the additional off-label application of an ultrasound contrast agent [9]. However, all described techniques have so far primarily been investigated in adults. A recently published small case series in pediatric patients has suggested that, due to small lymph node diameters in children, confirmation by contrast-enhanced ultrasound (CEUS) is especially useful [10]. Nevertheless, while CEUS seems to be a viable tool for needle position confirmation for DCMRL in general, the question arises as to whether the additional off-label ultrasound contrast application is necessary—especially in children [10, 11].

The aim of the present study was therefore to evaluate the success rate of ultrasound-guided injection needle position confirmation for DCMRL by application of only physiological saline solution in a large pediatric patient cohort.

## Material and methods

### Patient cohort

For this retrospective study, consecutive pediatric patients undergoing nodal DCMRL with ultrasound-guided needle positioning due to clinically suspected lymphatic pathologies were included between 05/2020 and 12/2022. DCMRL was part of the in-house standard clinical work-up in this patient cohort. Patients and/or their parents were informed in detail about the clinical DCMRL procedure and provided written informed consent. This included especially the off-label use of the MR contrast agent for lymphangiography. The institutional review board approved the data analysis and waived additional informed patient consent for retrospective data analysis.

### MR-lymphangiography

DCMRL was performed on a 3-T system (Ingenia Elition; Philips Healthcare) with a detachable MR table as described before [9]. One hundred seven out of 116 (92.2%) examinations took place under deep sedation. Patients were placed in a supine position on the detachable MR table and initially underwent non-contrast imaging including T2-weighted imaging and non-contrast T1-weighted imaging. Thereafter, the patient was transferred from the scanner room to a nearby ultrasound unit and the groins were disinfected and sterilely covered for the subsequent sterile puncture using also a sterile cover for the ultrasound probe.

A 22-gauge standard steel spinal needle (BD Medical) was connected to extension tubing with a three-way stopcock. This was used to connect a 10 mL syringe containing diluted gadobutrol (Gadovist, Bayer Healthcare), with a concentration of 1.0 mmol/mL diluted 1:4 with physiological saline solution, as well as another 10 mL syringe with physiological saline solution.

**Fig. 1:**
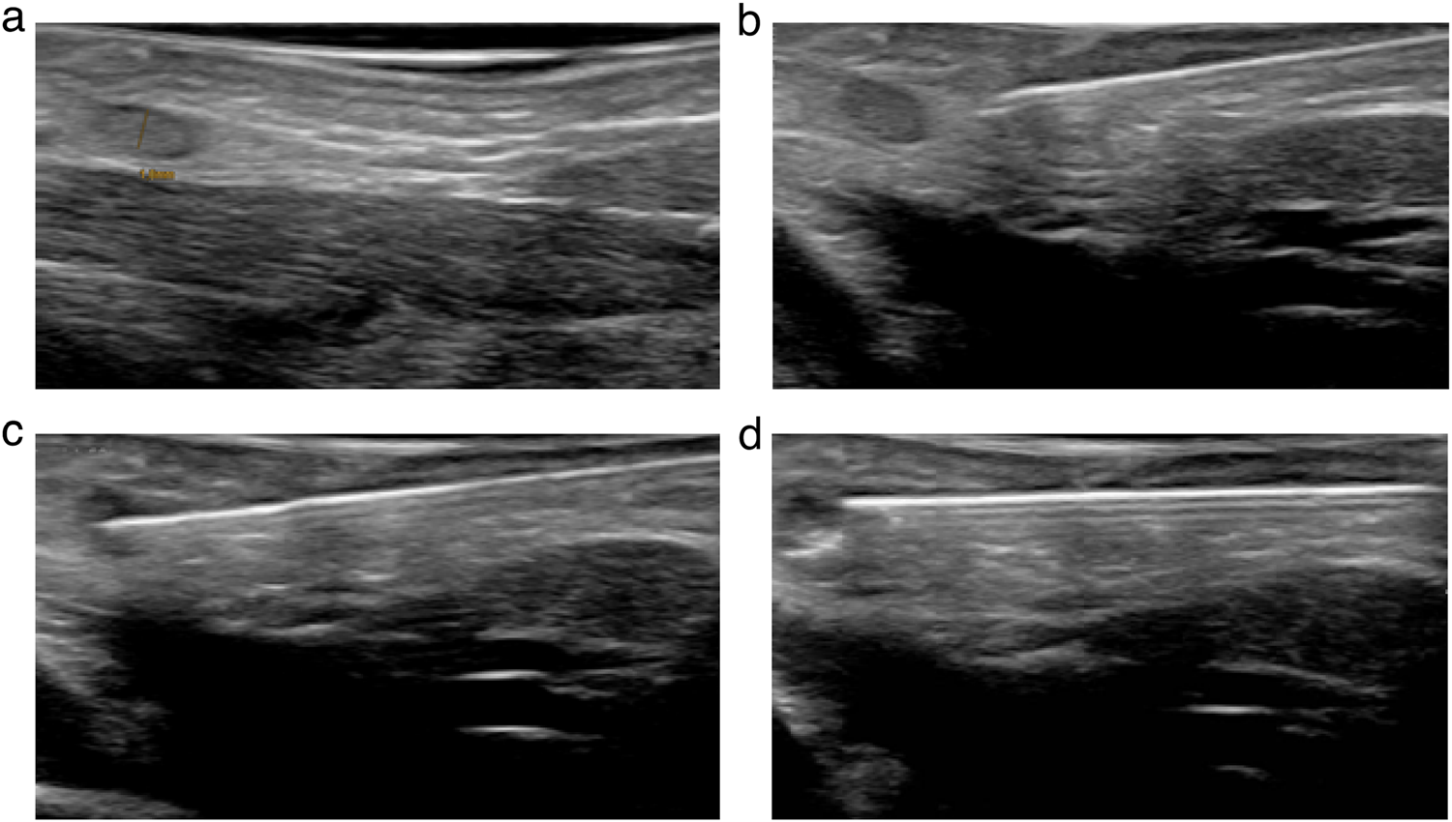
Sonographically guided inguinal lymph node puncture using a linear 18 MHz probe. After identification of a node with a diameter of 1.8 mm (**a**), a 22-gauge needle is advanced (**b**), and the lymph node is punctured (**c**) with subsequent injection of a small amount of saline solution and consecutive distension (**d**)

Thereafter, both groins were sonographically evaluated for target inguinal lymph nodes using a linear 18 MHz probe (Affinity 50, Philips Healthcare) (Fig. 1a). Considering the often small lymph node diameters, especially in infants, no lower cut-off diameter was defined to identify lymph nodes suitable for puncture. All ultrasound-guided lymph node punctures were performed by the same interventional radiologist (C.C.P. with 13 years of interventional experience) as described in detail before [9, 12].

After lymph node identification, a needle was advanced percutaneously at a shallow angle toward the selected node (Fig. 1b). The needle tip then was positioned in the transitional zone of the node between the cortex and hilum (Fig. 1c). To confirm the correct placement of the needle within the lymph node, 0.5–1 mL of physiological saline-solution was injected slowly, by hand, during continuous ultrasound-imaging. When the needle was correctly positioned, an expansion of the lymph node (i.e., any increase in its size) was observed (Fig. 1d). If no expansion was noted, or if any leakage into the surrounding tissue was detected, immediate repositioning of the needle was undertaken. Leakage of saline solution around the lymph node presents as hypoechoic strands appearing in the fatty tissue on ultrasound comparable to the presentation of subcutaneous edema. This could involve either adjusting the position of the needle tip by 1–2 mm, placing the needle in a different part of the same lymph node, or in a different lymph node.

**Fig. 2:**
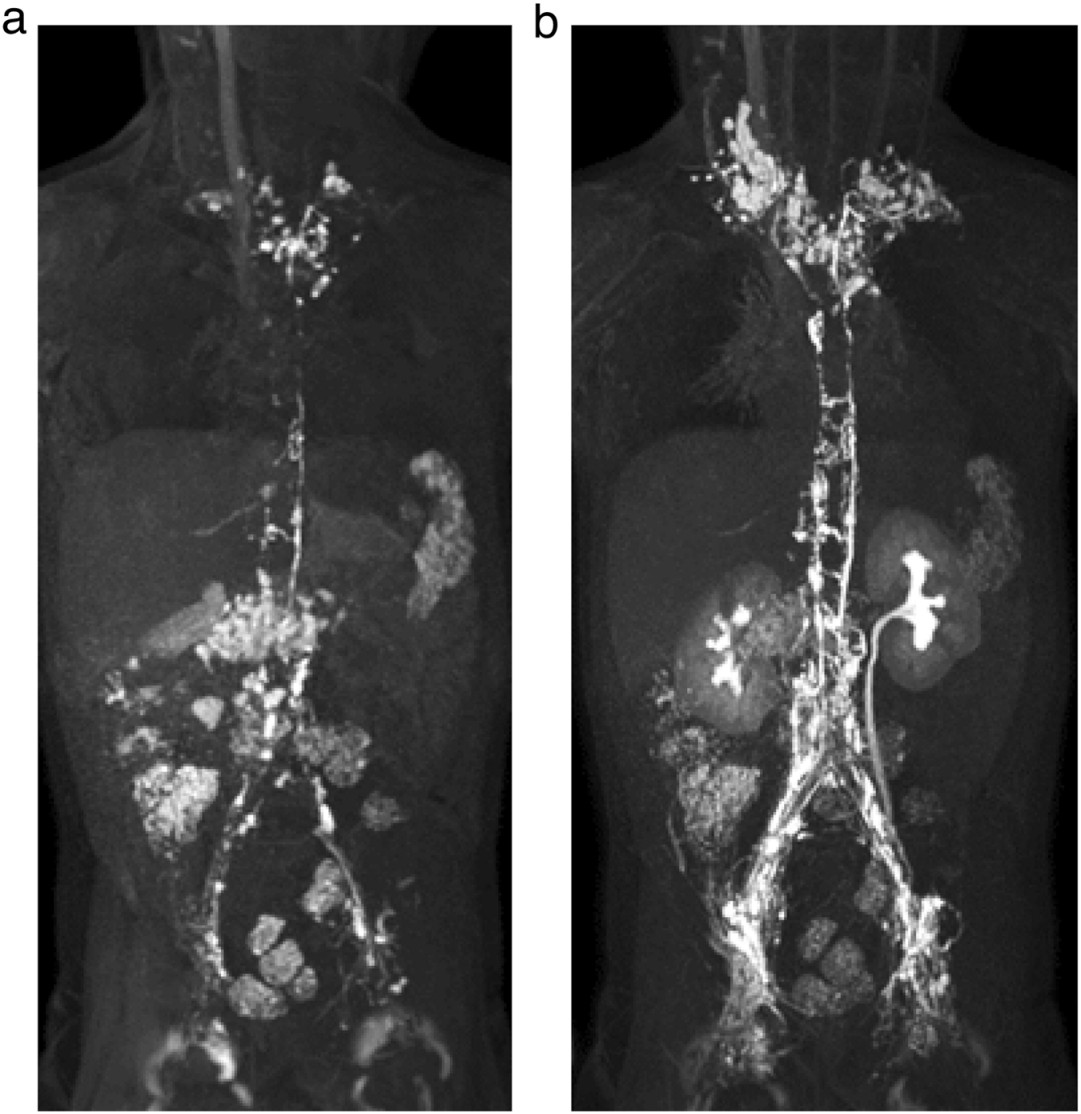
Maximum intensity projections (MIPs) in the coronal plane of T1-weighted, contrast-enhanced MR-lymphangiograms of a 5-year-old boy with univentricular heart disease and Fontan palliation suffering from intermittent plastic bronchitis DCMRL shows a typical central lymph flow abnormality with reflux into mediastinal and cervical as well to a lesser extent into peribronchial lymphatics (**a**, **b**). There is a minor venous enhancement not limiting lymphatic assessment (**b**)

Once the needle was satisfactorily positioned, the patient was transferred back into the MR scanner. DCMRL was conducted with a slow and continuous infusion of the diluted contrast agent by hand with a flow rate of 1 mL/min (Fig. 2a, b). During contrast application, a coronal 3DT1-weighted multi-echo gradient-echo (mDIXON) sequence was repeatedly acquired at 1-min intervals (TR 4.2 ms, TE 1.47 ms and 2.7 ms, flip angle 20°, field of view: 400 mm, matrix: 400 × 400 mm, slice thickness: 1 mm, acquisition time 49.1 s). Contrast distribution was observed on these dynamic images during the entire examination.

Needle repositioning was performed when either no enhancement of lymphatic vessels, or only venous enhancement, was seen. This repositioning could involve either retracting the needle tip by 1–2 mm while continuing with the MR examination (referred to as “in-bore needle retraction”) or secondary ultrasound-guided repositioning. If ultrasound-guided repositioning was required, it was performed back in the ultrasonography room.

### Data acquisition and definitions

Clinical and procedural data were collected from the electronic patient files. Ultrasound images and MR-lymphangiograms were gathered from the radiological picture archiving system. Documented sonographic images were used to identify the rate of groin lymph node detection, as well as to measure the short diameter of the punctured lymph node prior to saline injection. On injection of saline solution, a needle position within the node was confirmed by any lymph node distension without leakage into the adjacent tissue visible on ultrasound. In the event of a leakage, fluid pathways around the lymph node in the fatty tissue can be seen on ultrasound as hypoechoic stranding.

**Fig. 3:**
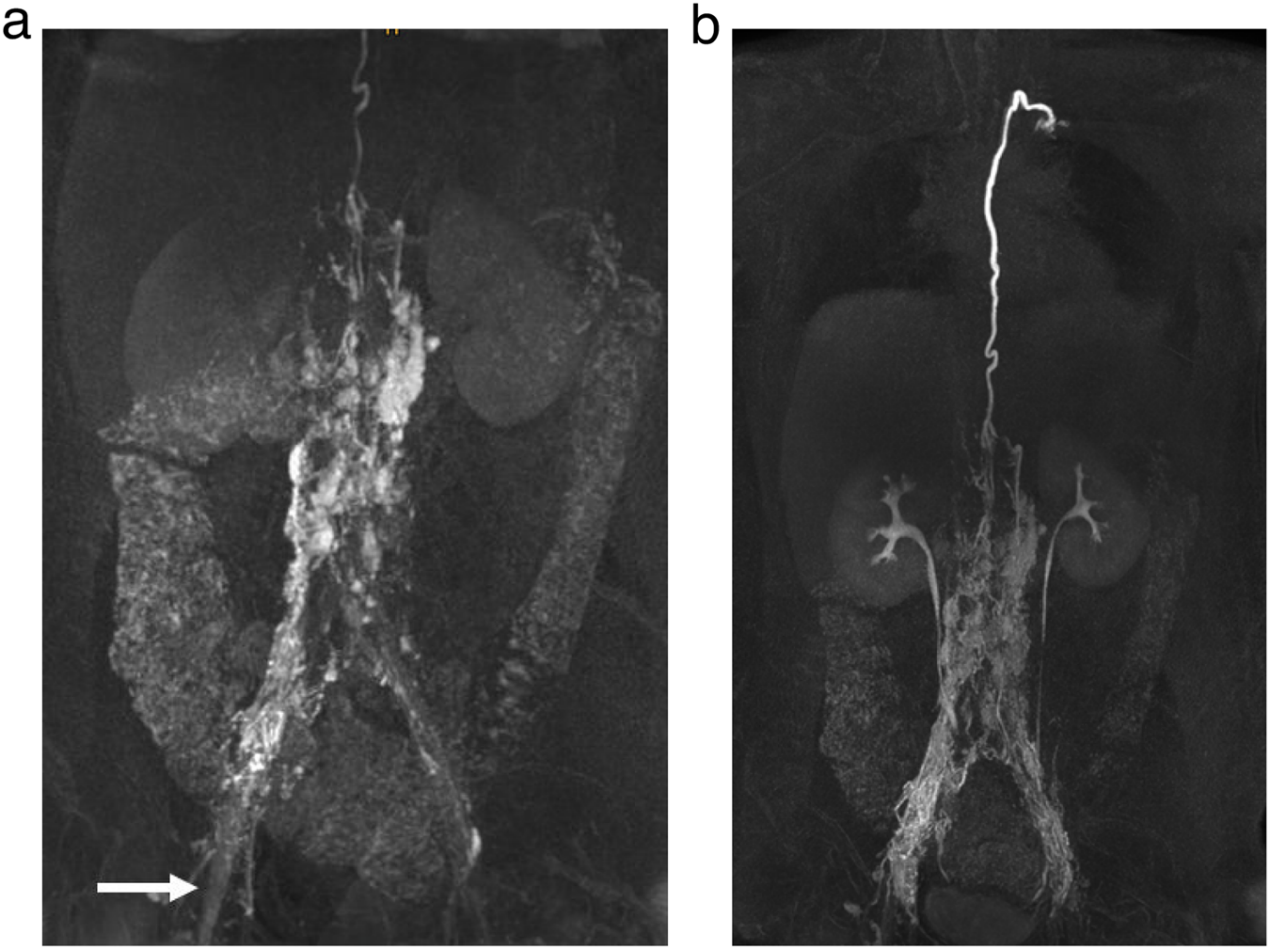
Maximum intensity projections (MIPs) in the coronal plane of T1-weighted, contrast-enhanced MR-lymphangiograms at different time points of a 14-year-old girl with Fontan circulation and protein-losing enteropathy. Central lymphatic enhancement via bilateral inguinal lymph nodes is evident in both the early (**a**) and late phase (**b**) images. While on the left there is enhancement of only lymphatic vessels and nodes, the right iliac vein shows minimal venous enhancement (white arrow) originating from the nodal injection site

Subsequent MR-lymphangiograms were reviewed by two radiologists in consensus (C.C.P. and J.W. with 13 years and 8 years of experience, respectively) regarding enhancement of iliac, retroperitoneal and thoracic lymph vessels and nodes and/or a lymphatic pathology. Venous enhancement of iliac veins and inferior vena cava as signs of venous drainage from the punctured lymph nodes were noted and graded as either minimal or marked venous run-off (Fig. 3a, b). Venous run-off was graded as minimal when faint enhancement of the vein was visible not obscuring adjacent lymphatic structures and as marked when primarily a strong venous enhancement was seen. Necessary needle repositioning was recorded as either in-bore needle retraction or ultrasound-guided repositioning.

**Fig. 4:**
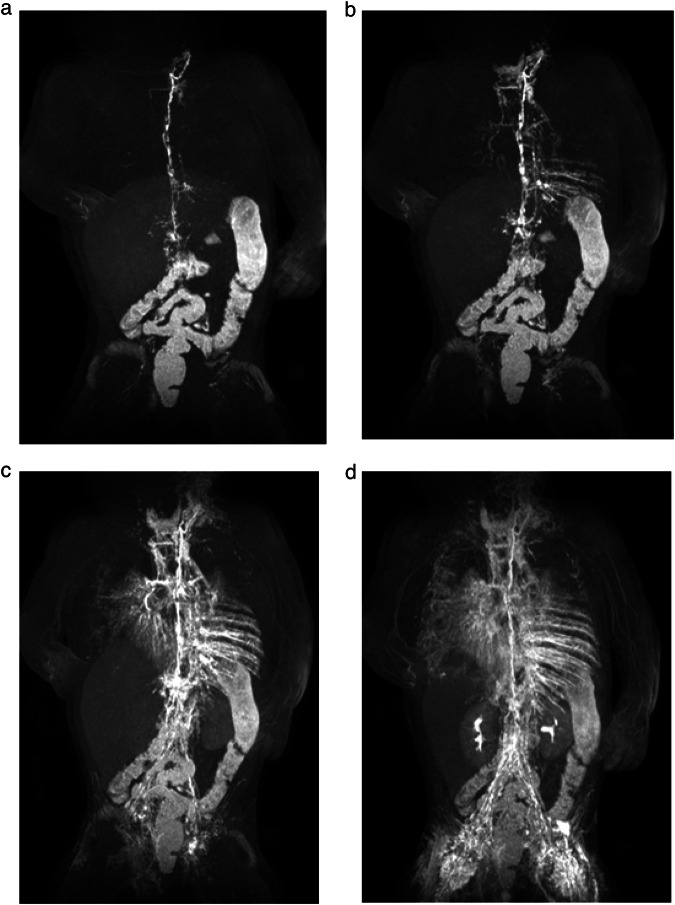
Maximum intensity projections (MIPs) in the coronal plane of T1-weighted, contrast-enhanced MR-lymphangiograms at different time points of a 5-month-old girl with lymphatic drainage disorder with massive chylolymphatic reflux in Noonan syndrome. The thoracic duct drains into a strongly refluxive lymphatic vessel in the mediastinum (**a**, **b**). Reflux via strong intercostal lymphatic vessels into the left dorsal thoracic wall (**b**, **c**). Strong reflux into the right dorsal abdominal wall, the left retroperitoneum, the lungs, the mediastinum, and the soft tissues of the neck (**c**). Overall delayed, but in the late phase preserved, venous contrast with renal excretion of the contrast medium (**d**)

When the respective ipsilateral iliac lymph vessels demonstrated enhancement on MR contrast-agent injection, this was defined as the primary technical success of DCMRL (Fig. 4a-d). This included cases with minimal venous enhancement originating from the injection site not obscuring lymphatic enhancement (Fig. 3a, b). Secondary technical success was defined as the successful enhancement of iliac lymphatics after at least one needle repositioning (either in-bore needle retraction or ultrasound-guided repositioning). The DCMRL examination was considered clinically successful when enhancement of the central lymphatic system (retroperitoneal and/or the thoracic duct) and/or a lymphatic pathology was observed.

Finally, peri-interventional complications were noted if present.

### Statistical analysis

Descriptive statistics were performed for patient characteristics and imaging findings. Data are given as mean ± standard deviation for continuous variables, median and range for skewed continuous variables or count for categorical variables.

## Results

90 consecutive pediatric patients (32 female, 58 male; mean age at first examination 6.1 years ± 5.5 years, range 3 weeks to 17.7 years) with clinically suspected lymphatic abnormalities were included in the study (see Table 1 for details). Overall 116 DCMRL examinations were performed. Some patients were examined more than once during the course of the disease to assess treatment effects. 71/90 (78.9%) patients underwent one, 12 (13.3%) patients two, and 7 (7.8%) patients three DCMRLs during the study period.

**Table 1:** Patient characteristics

Variable	Value
Number of patients	90
Number of investigations	116
Investigations under anesthesia/sedation	107
Sex	
Male	58
Female	32
Mean age at first examination (years ± SD)	6.1 ± 5.5
Age at magnetic resonance imaging	
< 1 year	28
1–5 years	40
5–10 years	21
10–18 years	27
Mean body size (cm ± SD)	102 ± 35
Mean body weight (kg ± SD)	18.6 ± 16
Mean lymph node diameter (mm ± SD)	2.5 ± 1.2
Clinical indication for MR-lymphangiography	
Chylothorax	40
Chylous ascites	5
Combined chylothorax/chylous ascites	3
Post-Fontan patients with a history of lymphatic complications	19
Bronchitis plastica	15
Protein-losing enteropathy	13
Localized lymphedema (e.g., genital)	10
Lymphatic malformations	5
Chylothorax + bronchitis plastica	3
Bronchitis plastica + protein-losing enteropathy	1
Leakage/lymphocele	1
Chylothorax + chylopericardium	1

At the time of the DCMRL 28/116 (24.1%) patients were < 1 year, 40/116 (34.5%) were between 1 year and 5 years, 21/116 (18.1%) were 6–10 years and 27/116 (23.3%) were between 11 years and 18 years. The mean body weight was 18.6 kg, ranging from 1.5 kg to 73 kg.

Results of ultrasound-guided needle placement and DCMRL examinations and their interrelations are summarized in Tables 2 and 3.

**Table 2:** Results of ultrasound-guided needle placement and MR contrast medium injection

	Number of groins
Total	232
Ultrasound needle puncture results	
Lymph nodes detectable	232
Attempted nodal needle placements	230
Lymph node distension	230/230 (100%)
MRL results	
Nodal contrast medium injections	230
Accompanying venous enhancement	5/230 (2.2%)
Technical success	229/230 (99.6%)
Primary success	228/230 (99.2%)
Secondary success	1/230 (0.4%)
No technical success	1/230 (0.4%)
Overall clinical success	229/230 (99.6%)

Primary success: immediate enhancement of iliac lymphatics; secondary success: enhancement of iliac lymphatics after at least one needle repositioning

**Table 3:** Interrelations between lymph node distension on saline injection on ultrasound and primary technical success of MR-contrast injection

	Lymphatic enhancement on MRL	No lymphatic enhancement on MRL
Lymph node distension in the US	228 (99.2%)	2 (0.8%)
No lymph node distension on US	0	0

For these 116 examinations, 232 groins were sonographically evaluated for target lymph nodes. Lymph nodes were identified in all 232/232 (100%) groins. The short axis diameter of the target lymph nodes ranged from 1 mm to 5 mm (mean 2.5 ± 1.2 mm). A lymph node diameter of only 1 mm was observed in 24 patients (26.7%).

Subsequent ultrasound-guided needle placement was attempted in 230/232 groins (99.1%) due to local skin inflammation in two groins. It was therefore attempted to establish bilateral nodal access for 114/116 DCMRLs (98.3%), while for the remaining 2/116 (1.7%) examinations only a unilateral access was attempted. After needle-placement lymph node distension was seen on ultrasound upon saline injection in all 230/230 (100%) groins.

Thereafter the patient was transferred back into the MR scanner and all 230 punctured lymph nodes were injected with MR contrast-agent as described above. Overall 229/230 (99.6%) nodal contrast applications were technically successful. In 228/230 lymph nodes (99.2%) MR showed a prompt and adequate enhancement of iliac lymphatics (primary technical success). Accompanying minimal venous enhancement originating from the punctured lymph node was seen in 5/230 groins (2.2%) without, however, obscuring lymphatic structures (Fig. 3a, b).

**Fig. 5:**
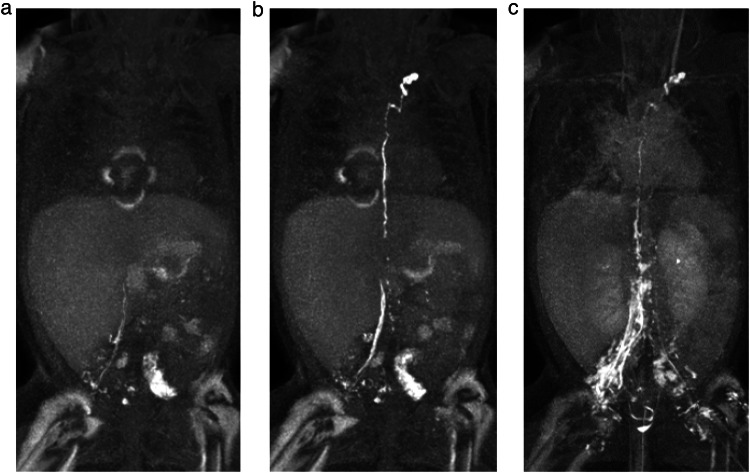
Maximum intensity projections (MIPs) in the coronal plane of T1-weighted, contrast-enhanced MR-lymphangiograms at different time points of a 2-year-old boy with suspected central lymphatic flow anomaly and chylous ascites. **a**, **b** Demonstrate central lymphatic enhancement via a right inguinal lymph node. There is no visible enhancement of left iliac lymphatics. After in-bore needle retraction in the left groin by 1 mm, run-off of contrast agent can be seen also in left iliac lymphatics (**c**) (secondary technical success)

**Fig. 6:**
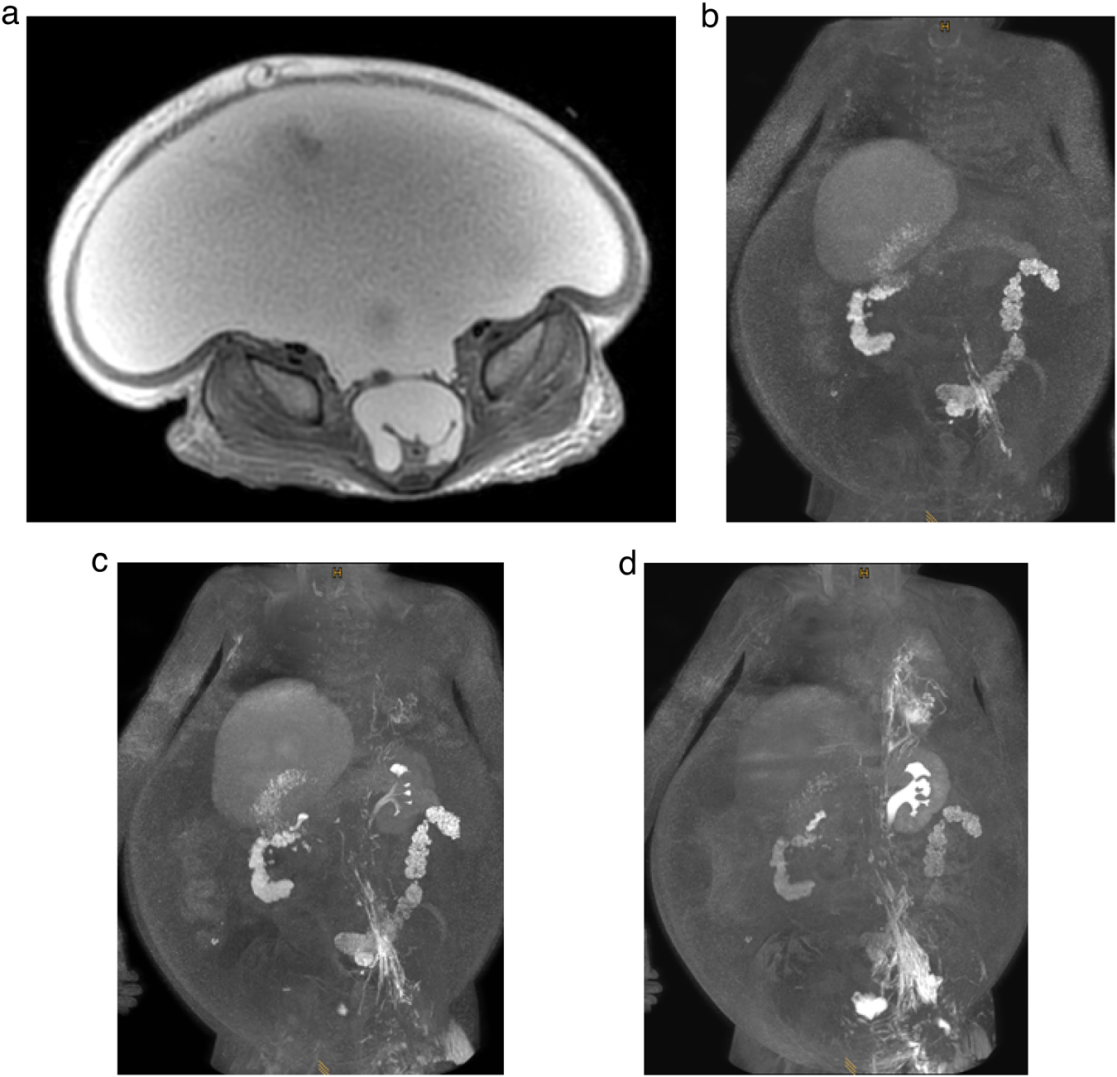
Axial fat-suppressed T2-weighted MR-image of a 6-year-old girl with extensive chylous ascites (**a**). Coronal Maximum intensity projections (MIPs) of T1-weighted, contrast-enhanced MR-lymphangiograms at different time points (**b**–**d**). No lymphatic enhancement was seen on the right side despite lymph node distension on saline injection and no lymph vessel enhancement was achieved despite needle retraction, as well as ultrasound-guided needle repositioning (**b**). However, as lymphatic run-off was seen on the contralateral side in this case, DCMRL was still clinically successful showing an extensive central lymphatic flow abnormality without a detectable thoracic duct and reflux into left-sided pulmonary lymphatics (**c**, **d**)

In 2/230 lymph nodes (0.8%), no lymphatic enhancement was seen despite lymph node distension on saline injection. In one of these nodes, in-bore needle retraction by 1 mm subsequently led to lymphatic enhancement (secondary technical success) (Fig. 5a–c). In the other lymph node, no lymph vessel enhancement was achieved despite needle retraction, as well as ultrasound-guided needle repositioning. However, as lymphatic run-off was seen on the contralateral side in this case, DCMRL was still clinically successful showing an extensive central lymphatic flow abnormality without a detectable thoracic duct (Fig. 6a–d).

Overall, all 116/116 DCMRL examinations (100%) were clinically successful showing enhancement of the central lymphatic system (retroperitoneal/thoracic lymphatics) and/or a lymphatic abnormality.

No complications associated with lymph node puncture, or saline or contrast injection were observed within a follow-up period of at least four weeks.

## Discussion

Currently, the most versatile technique for lymphatic imaging is DCMRL [13–15]. It allows for visualization of the central lymphatic system with high spatial and time resolution and therefore, enables the assessment of a wide spectrum of lymphatic conditions from simple lymphatic leakages to complex lymphatic flow abnormalities and malformations [1, 7, 12, 16–22]. As contrast-agent application for DCMRL is performed through lymph node accesses after ultrasound-guided cannulation, a correct and stable needle position within a lymph node is of paramount importance to ensure adequate enhancement of the lymphatic system. This poses a technical and logistical challenge, especially in children and infants in whom lymph nodes usually are small and DCMRL often has to be performed under sedation or general anesthesia [7–9] limiting possible transfers in and out of the MR-scanner for ultrasound-guided needle position revision. The possible number of transfers is limited in these cases, as the required anesthesia unit and accompanying tubing must be transported as well, avoiding any material dislocation thereby increasing logistical challenges and time consumption for every transfer.

As the initial step for successful MRL, groin lymph nodes for needle placement have to be identified on ultrasound. This can be performed as a separate ultrasound examination in the days or weeks prior to a scheduled DCMRL examination or during the actual DCMRL examination [9, 12]. However, there is, so far, no general consensus on a minimum lymph node size suitable for puncture. In a recent small series in pediatric patients [10], a minimum short lymph node diameter of 2 mm was used while the lowest lymph node diameter in a previous work of Wagenpfeil and colleagues was 1 mm [9]. In the present cohort, no minimum diameter was defined and therefore the mean diameter was as low as 2.5 mm with over 25% of patients presenting with only 1 mm lymph nodes. The technical and clinical success of DCMRL was still nearly perfect despite these very small access lymph nodes. As our experience demonstrates that DCMRL is successful in the vast majority of patients even without a predetermined minimum node diameter, sonographic evaluation in advance of the actual DCMRL is not strictly necessary, making logistics easier. Furthermore, over 25% of patients in the present study would otherwise have been excluded from DCMRL when employing the minimum lymph node diameter cut-off suggested by Fung et al. It needs to be noted, however, that our institution is a tertiary reference center for lymphatic imaging and interventions and that the interventionalist has over 13 years of experience with these technique.

Apart from the anatomical conditions in the groins of the patients, the technique of confirming accurate needle placement has recently been a matter of some discussion [11, 23]. Initial reports described confirmation by injection of water-soluble X-ray contrast agent under fluoroscopy [1, 3]. However, as this technique requires a combined XMR-suite, its application is limited. Nadolski and colleagues subsequently published their initial experiences with needle position validation by CEUS in adults, obviating the need for an XMR suite [8].

Fung et al demonstrated the feasibility of this CEUS approach also in pediatric patients [10]. They performed needle placement in groin lymph nodes in seven children with bilateral inguinal nodal access (14 punctured nodes) using a comparable puncture technique as in the present study with the patients also on a detachable MR-table [8, 10]. Included patients had a median age of 13 years (interquartile range 3.5–7.5 years) with attempted punctures of lymph nodes larger than 2 mm in diameter [10]. An ultrasound contrast agent was used to visualize efferent lymphatics as a sign of adequate needle positioning. After ultrasound contrast injection needle repositioning was necessary in 2/14 groins. A cannulation success rate of 12/14 nodes (85.7%) was reported. The subsequent MR contrast injection, was successful in all of these 12 cases [10]. However, although CEUS seems to be a viable tool for needle position confirmation for DCMRL, the question arises as to whether this additional off-label ultrasound contrast application is really necessary—especiallywhen examining children. Although very rare, side effects associated with ultrasound contrast agents, as well as the additional costs for the contrast agent also have to be considered [24].

The largest published series to date on the technical success of nodal DCMRL including 171 punctured lymph nodes in a primarily adult patient cohort demonstrated that the use of only saline solution instead of an ultrasound contrast agent is sufficient for needle position verification [9]. Overall technical success was observed in 169/171 lymph node punctures (98.8%). Primarily venous run-off was observed in only 6/171 lymph nodes (3.5%) on DCMRL and was resolved by minimal needle retraction in the scanner.

In the present study, we have investigated whether ultrasound-guided injection of physiologic saline provides a reliable assessment of correct needle placement in a large pediatric population. The main finding of this study was that lymph node distension is a reliable sign of correct needle placement. In 228/230 cases with lymph node expansion on saline-solution, adequate lymphatic run-off of MRcontrast-agent was demonstrated. In one additional case (1/230), only minimal retraction of the needle was required to achieve adequate lymphatic enhancement on DCMRL.

A varying degree of venous enhancement was observed in 5/230 punctures originating from the injection site, but necessitated needle repositioning in only 1/230 of cases. A possible explanation for venous enhancement may be lymph-venous shunts at the level of the lymph node as described by Kariya et al [25]. However, marked venous enhancement without lymphatic enhancement may also result from a central needle position in the lymph node hilum. Therefore, positioning the needle tip at the corticomedullary junction of the lymph node has been recommended [2, 4]. In contrast to saline injection, an ultrasound contrast agent used for needle position validation may be advantageous in showing primary venous run-off already before MR contrast-agent injection [7, 23]. This was demonstrated by Nadolski et al in 1/28 cases with enhancement of the femoral vein on ultrasound contrast injection [8]. However, primary venous run-off (venous run-off initially occurred before the enhancement of the lymphatic system) was only visible in 1/230 cases in our cohort and could be resolved in this case by simple needle retraction inside the scanner without the need for repeated ultrasound.

Interestingly, the initial report by Nadolski and colleagues on CEUS demonstrated necessary needle repositioning in 6/28 cases (21.4%), pointing to the overall usefulness of the technique as in these cases needle repositioning seems to have been necessary for a successful DCMRL [8]. However, rates of necessary needle repositioning in the report by Wagenpfeil et al in adults, as well as in the present report in pediatric patients both employing only saline injection were considerably lower (4.6% and 0.8%, respectively). A possible explanation may be that CEUS is able to detect smaller leakages of contrast-agent around the lymph node, as well as venous runoff with a higher sensitivity than plain saline injection. This subsequently may prompt repositioning of the needles. However, these repositioning procedures may ultimately not have been necessary for a successful DCMRL as smaller leakages and/or shunts not seen on saline-solution injection do not seem to play a clinically relevant role.

Finally, it is important to note that the application of nodal ultrasound contrast injection may be an interesting tool to assess efferent lymphatic flow originating from the lymph node or to even enable assessment of the lymph-venous junction [10, 23]. However, in our experience, the application of ultrasound contrast medium purely for sonographic evaluation of a proper needle position for DCMRL is not necessary.

This study has several limitations. First, it is important to note that this was a retrospective study on clinical DCMRL examinations with inherent methodological limitations, e.g., partially incomplete clinical documentation of patients’ prior history in the clinical database or availability of only some saved still ultrasound images which prevented the measurement of distended lymph node diameter. Furthermore, as the examinations were conducted in a clinical setting with varying lymph node sizes, no precise definition of acceptable distension of the punctured lymph node on saline injection was employed. Any visible increase in the size of the node on injection without leakage into the surrounding tissue was accepted to demonstrate an intranodal position of the needle tip. Second, the research was conducted at a single medical center, and all lymph node punctures were performed by the same experienced interventional radiologist, somewhat limiting the generalizability of the described success rates. Further investigations into technical and clinical success rates at different centers are certainly warranted. Third, the primary focus of this study was on ensuring correct needle placement and did not address the technique for securely transferring the patient from ultrasound back into the MR-scanner without the risk of needle dislocation. All DCMRLs were conducted using an MR scanner equipped with a detachable table, which allowed for minimal patient movement during the transfer in and out of the scanner. Different approaches might be necessary if the examination table cannot be moved in this manner. Additionally, it is important to acknowledge that lymph node diameter measurements could only be taken from the documented sonographic images, potentially leading to variations in the lymph node axis between different cases. As no additional imaging techniques demonstrate lymphatic run-off from the punctured lymph node other than the MRL itself when employed in this cohort of patients, the observed 2/230 primary technical failures of MR-contrast injection despite visible lymph node distension on ultrasound ultimately remain unclear. In one of these two cases, secondary needle movement was the most likely cause for initial technical failure as slight retraction of the needle led to technical success. In the other case also repeated needle positioning did not lead to lymphatic enhancement, making a lack of lymphatic run-off from the punctured lymph node the most likely cause of failure. Further investigation into lymphatic run-off rates from the punctured lymph node without confounders such as patient movement into an MR-scanner would be interesting in this respect.

In conclusion, using plain saline solution to confirm sonographically placed intranodal needle position for DCMRL is a very reliable and safe technique with a very high success rate of lymphatic enhancement in pediatric patients. The additional off-label application of ultrasound contrast-agent may not be necessary in a clinical setting but further comparative study is needed.

## Abbreviations

CEUS: Contrast-enhanced ultrasound
DCMRL: Dynamic contrast-enhanced MRL
MR: Magnetic resonance
MRL: Magnetic resonance lymphangiography
XMR: Angio-MR
